# Electro-optical effects of organic N-benzyl-2-methyl-4-nitroaniline dispersion in nematic liquid crystals

**DOI:** 10.1038/s41598-020-71306-1

**Published:** 2020-08-31

**Authors:** Pravinraj Selvaraj, Karthick Subramani, Brahadeeswaran Srinivasan, Che-Ju Hsu, Chi-Yen Huang

**Affiliations:** 1 Department of Physics, National Changhua University of Education, Changhua, 500 Taiwan; 2 Department of Physics, University College of Engineering, BIT- Campus, Anna University, Tiruchirappalli, 620 024 India; 3 Graduate Institute of Photonics, National Changhua University of Education, Changhua, 500 Taiwan

**Keywords:** Displays, Liquid crystals

## Abstract

The dispersion of organic N-benzyl-2-methyl-4-nitroaniline (BNA) in nematic liquid crystals (LCs) is studied. BNA doping decreases the threshold voltage of cell because of the reduced splay elastic constant and increased dielectric anisotropy of the LC mixture. When operated in the high voltage difference condition, the BNA-doped LC cell has a fall time that is five times faster than that of the pure one because of the decrements in the threshold voltage of the cell and rotational viscosity of the LC mixture. The additional restoring force induced by the BNA’s spontaneous polarization electric field (SPEF) also assists to decrease the fall time of the LC cell. The decreased viscosity can be deduced from the decrements in phase transition temperature and associated order parameter of the LC mixture. Density functional theory calculation demonstrates that the BNA dopant strengthens the absorbance for blue light, enhances the molecular interaction energy and dipole moment, decreases the molecular energy gap, and thus increases the permittivity of the LC mixture. The calculation also shows that the increased dipole moment, polarizability, and polarizability anisotropy increase the dielectric anisotropy of the LC mixture, which agrees with the experimental results well. BNA doping has a promising application to the fields of LC devices and displays.

## Introduction

Nematic liquid crystals (LCs) are extraordinarily responsive and optically uniaxial materials. Nematic LCs have been extensively used in diverse applications, such as optical nonlinearity, optical phase modulators, micro-displays, flat panel displays, optical antennas, and optical switching, due to their electro-optical property and other admirable features^1–5^. LCs with a fast response are vital in removing motion blur in moving pictures and resolving cross-talk in 3D displays^6–9^. The response time of LCs should be less than 3 ms to diminish motion blur and cross-talk. Several techniques have been proposed to resolve this issue, and they include tuning the viscosity of materials^10^, varying the anchoring energy^11^, changing the electrode shape and driving scheme^12^, changing the cell gap^13^, modifying the guest–host material^14^, and applying new switching modes^15^.

The improvements in the electro-optical properties of LCs with the dispersion of different gust entities were presented recently. For example, Y. Dai et al. demonstrated that the incorporation of γ-Fe_2_O_3_ nanoparticles into LCs results in a response time of 4.75 ms with the application of the overdriving scheme, which is three times faster than pure LCs^16^. A response time of around 4 ms was obtained with the addition of a small amount of dye to a polymer-dispersed liquid crystal (PDLC) or functionalized carbon nanotubes dispersed in optically isotropic LCs. However, in this case, the excellent response speed is valid only for operation at a relatively high voltage^17,18^. Blue-phase LCs have a response time of 0.5 ms, but they still have drawbacks, such as hysteresis, high operation voltage, and narrow temperature range^19^.

N-Benzyl-2-methyl-4-nitroaniline (BNA) is a polar N-derivative 2-methyl-4-nitroaniline (MNA) with a high electro-optical capability due to its relatively high nonlinear optics (NLO) coefficient (234 ± 31 pm/V) compared with other organic NLO materials^20^. It was developed by the Hashimoto group^21^. BNA belongs to an orthorhombic space group of Pna2_1_ and exhibits high second harmonic generation (SHG) efficiency due to its proper phase match, which is 300 times higher than that of standard urea^21,22^. BNA can also be utilized for the realization of devices with maximized THz, NLO, and piezoelectric properties. The angle between the two benzene rings of BNA is around 80° and results in its L shape^23^. The precise L-shape of the BNA molecule in orthorhombic crystal results in the establishment of two N–H^…^O hydrogen bonds (HBs) that enable intermolecular charge transfer (CT) to obtain high SHG efficiency^24,25^. These groups represent push–pull systems with intramolecular CT between the electron donor (-NH_2_) and acceptor (-NO_2_) by means of the conjugated benzene ring^26^. The nitro group in the BNA molecule plays an important role due to the establishment of HBs and contributes to fundamentals, overtones, and lattice vibration couplings with intermolecular CT^25^. Orthorhombic BNA obeys intermolecular and intramolecular CT that lie on the same plane but have opposite directions^27^. In our previous work, a BNA–LC mixture was used to fabricate a large-aperture hole-patterned LC lens to improve the response time for the first time^28^. The BNA-doped LC lens had a turn-off time that was ∼ 6 times faster than that of undoped LC lens because the BNA dopant decreased the rotational viscosity of the LC mixture. However, the important mechanism for the decrease in the rotational viscosity of LC mixtures with BNA doping has not been discussed in detail yet.

In the current study, we attempted to understand the effect of BNA doping on nematic LCs. The transmission spectrum of a BNA-doped LC cell was used to observe the absorbance caused by the BNA dopant. The dielectric spectrum and voltage-dependent transmission of the BNA-doped LC cell were measured to determine the threshold voltage of the cell and the dielectric anisotropy and birefringence of the LC mixture, and the results were used to calculate the splay elastic constant of the LC mixture. The phase transition temperature of the BNA-doped LC cell was observed to confirm the tendencies in the order parameter and splay elastic constant of the LC mixture. The decreased order parameter reduced the relaxation time and active energy and hence the rotational viscosity of the LC mixture. The response time of the BNA-doped LC cell was also measured and showed that the BNA-doped LC cell had a fivefold faster fall time than the pure one due to the decreased threshold voltage of the cell and rotational viscosity of the LC mixture and the additional restoring force by the BNA’s spontaneous polarization electric field (SPEF). Density functional theory (DFT) was utilized to demonstrate the molecular alignment geometry, polarizability, polarizability anisotropy, and dipole moment of the BNA-LC mixture and understand further the interactions between BNA and LC molecules. BNA doping increased the polarizability, polarizability anisotropy, dipole moment, and hence dielectric anisotropy of the LC mixture.

## Methods

**Figure 1:**
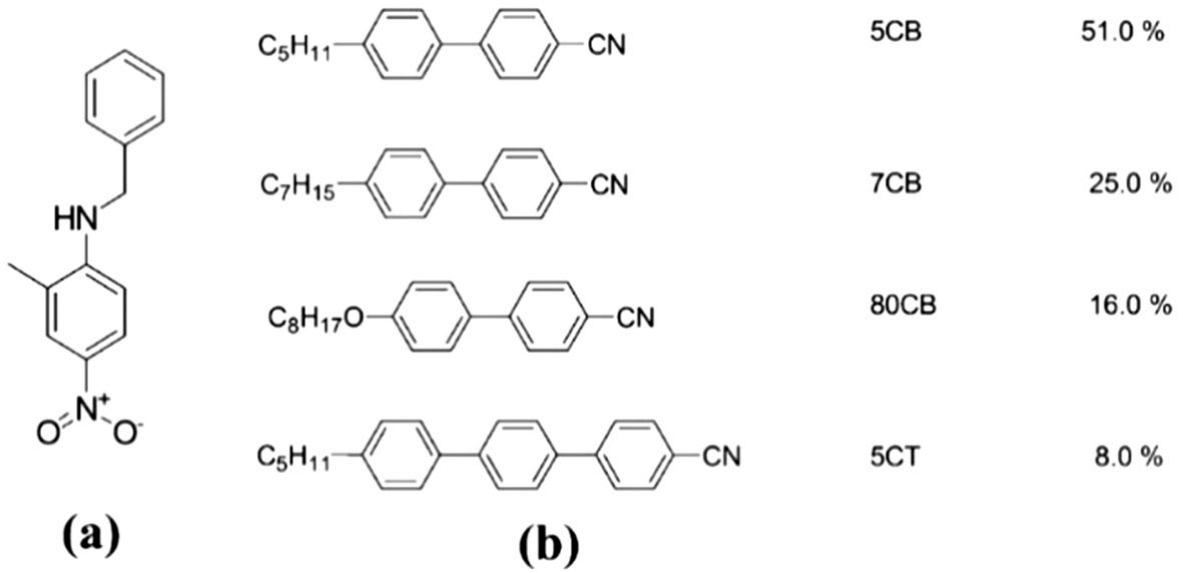
Molecular structure of (**a**) BNA and (**b**) E7 LC.

BNA was synthesized by adding commercially purchased reactants 2-methyl-4-nitroaniline (MNA) (20 g), hexamethyl phosphoric triamide (HMPA) (100 ml), sodium bicarbonate (22 g), and benzyl bromide (45 g) to a round-bottom flask, and the entire solution was refluxed for 30 h at 70 °C under a nitrogen atmosphere^21^. Subsequently, 500 ml of double-distilled water was added to the solution. The solution was extracted with diethyl ether and washed several times with saturated sodium chloride solution. The organic layer was then dried using anhydrous sodium sulfate powder, and diethyl ether was evaporated to obtain BNA powder (17 g, yield of 19.5%). The synthesized material was further purified by re-crystallizing it several times by using high-performance liquid chromatography (HPLC)-grade methanol as a solvent. The final compound yielded a single spot in silica-gel thin layer chromatography (TLC) (n-hexane: ethyl acetate = 7:3). The appearance of the synthesized BNA was yellow powder at room temperature (RT) and had a melting temperature of ~ 105 °C. Figure 1a shows the molecular structure of organic BNA.

Figure 1b shows the molecular structure of the nematic LC E7 (Daily Polymer Corp., Taiwan) used in the experiment. The nematic LC E7 was mainly composed of LC monomers 5CB, 7CB, 8OCB, and a small quantity of triphenyl. It had a nematic–isotropic phase transition temperature (*T*_*NI*_) of 64 °C, birefringence (*Δn*) of 0.22, rotational viscosity (*γ*) of 232.6 mPas, dielectric anisotropy (*Δε*) of 14.1, and elastic constant *K*_*11*_, *K*_*22*_, and *K*_*33*_ values of 11.1, 5.9, and 17.1 pN, respectively, at 20 °C. A commercial 5-μm-thick empty cell composed of two indium–tin–oxide (ITO) glass substrates was prepared. The inner surfaces of the substrates were coated with homogeneous polyimide and rubbed in the antiparallel direction. The thickness of the empty cell was confirmed with the interference method. An LC mixture consisting of organic BNA and nematic LC E7 was prepared, the BNA powder was directly dissolved in LCs without any solvents; then the mixture was ultrasonically stirred for 10 min at RT. The BNA concentration was set to 0.5, 1.0, 1.5, 2.0, and 3.0 wt%. The LC mixtures were heated to the isotropic phase to fill in the empty cell uniformly via capillary action and subsequently cooled down to the nematic phase.

The optical texture of the LC cell was determined using a polarizing optical microscope (POM) (DM EP, LEICA, Germany) to observe *T*_*NI*_. The LC cell was heated from the nematic to isotropic phase at a rate of 0.25 °C/min by using a temperature controller (T95-PE, Linkam, UK). The electro-optical properties of the BNA-doped LC cell were measured using the following setup. A He–Ne laser with 632 nm wavelength was used as incident light, and a BNA-doped LC cell was placed between a pair of crossed polarizers to obtain the voltage-dependent transmission (V–T). The rubbing direction of the cell had an angle of 45° with respect to the transmission axes of the polarizers. The pre-tilt angles of the BNA-doped LC cells were measured through the crystal rotation method^29^, which revealed that the angles were almost below 3°. The polar anchoring energy coefficients *W*_*polar*_ of the BNA-doped LC cells were estimated via the high electric field techniques^30^. The *W*_*polar*_ of the BNA-doped LC cells remained constant at ~ $1.3 \times 10^{ - 4}$ J/m^2^, as shown in Table 1. The dielectric spectra of the homogeneously aligned (HA) and vertically aligned (VA) BNA-doped LC cells were measured using an LCR meter (Hioki 3532-50, Japan) with an applied alternating current (AC) field of 0.01 V/μm to obtain permittivities that are perpendicular ($\varepsilon_{ \bot }$) and parallel ($\varepsilon_{\parallel }$) to the LC molecular axis, respectively. The *Δε* of the LCs was defined as the difference between $\varepsilon_{\parallel }$ and $\varepsilon_{ \bot }$ at a frequency of 1 kHz. The *Δn* of the LCs was derived with the phase retardation technique^5^.

**Table 1:** *W*_*polar*_ at various BNA concentrations.

BNA concentration (wt%)	0	0.5	1	1.5	2	3
*W*_*polar*_ (10^−4^ J/m^2^)	1.3	1.3	1.2	1.3	1.4	1.7

## Results and discussion

**Figure 2:**
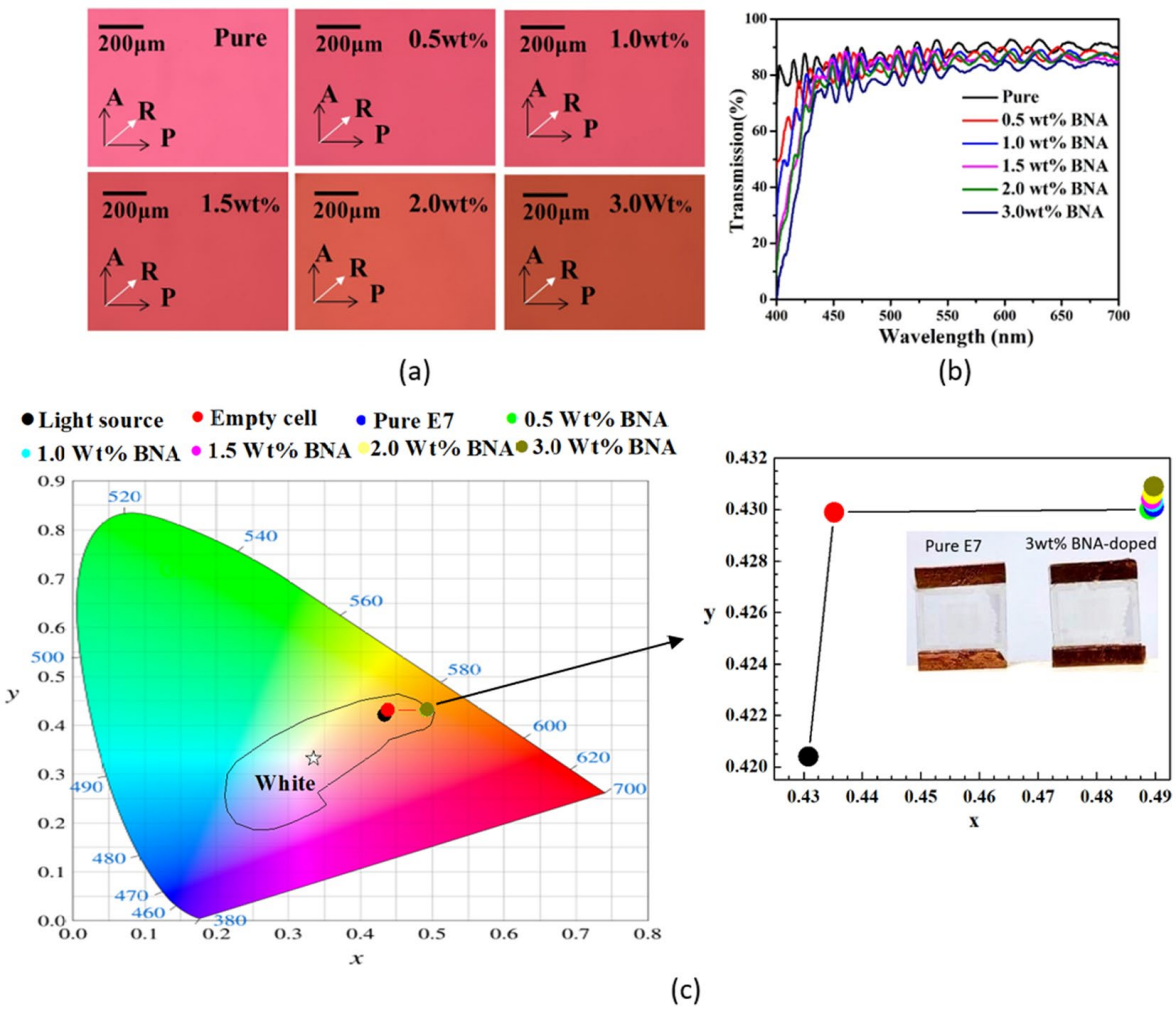
(**a**) POM photographs, (**b**) transmission spectra, and (**c**) CIE 1931 chromatographic coordinates of the BNA-doped LC cells. Inset shows the enlarged CIE chromatographic coordinates and the sample photos of the 3 wt% BNA-doped and pure E7 LC cells under daylight illumination. The solid arrows indicate the rubbing direction (R) and transmission axes of the polarizer (P) and analyzer (A).

Figure 2a shows the POM images of the BNA-doped LC cells, where the cell rubbing direction was placed at 45° with respect to the transmission axes of the polarizer and analyzer. The uniform colors throughout the cells confirmed that the LC molecules were aligned homogenously^31–34^. After BNA addition, we did not observe any significant defect aside from the slight color shift, indicating that BNA was well dispersed in the LC matrix and the *Δn* of the LC mixture slightly changed. Figure 2b presents the transmission spectra of the BNA-doped LC cells in the visible range between 400 and 700 nm. The light loss of around 12% (from 85 to 73%) was obtained because BNA doping changed the refraction index of the LC mixture and thus increased the refractive index mismatch among the surfaces between the glass substrates and LC layer. Notably, BNA has drastic absorbance at wavelengths less than 450 nm. Figure 2c shows a diagram of the International Commission on Illumination (CIE) 1931 chromatographic coordinates. It illustrates the stimuli-induced color change. Compared with the color coordinate of the used light source, those of the empty and BNA-doped LC cells exhibit slight shifts possibly because of absorbance by LC mixture and PI layers. The yellowish CIE chromatographic coordinates of the BNA-doped LC cells are related to the used light source and the short wavelength absorbance of the BNA doping (from Fig. 2b). The inset of Fig. 2c shows the enlarged CIE chromatographic coordinates and the sample photos, confirms that the small amount of BNA doping slightly contributes to the yellow tint in the CIE chromatographic coordinates. This experiment indicates that the BNA-doped LC cell successfully plays the role of a light intensity filter as it should be. For electro-optical components that require high color accuracy, more experiments are necessary for the collocation among light source, LC cell, and organic materials.

**Figure 3:**
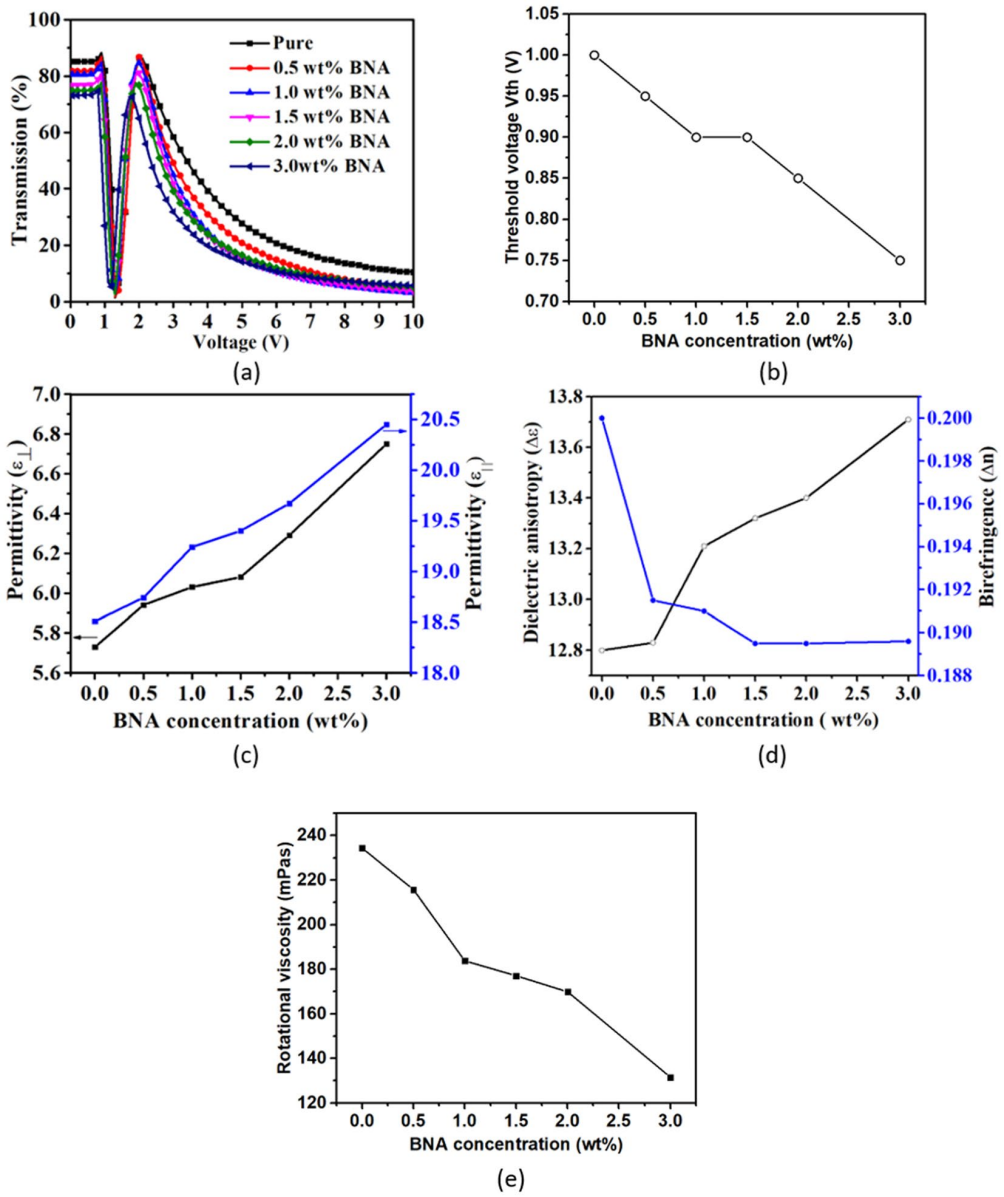
(**a**) V–T curves of the BNA-doped LC cells. (**b**) *V*_*th*_ as a function of BNA concentration. (**c**) $\varepsilon_{ \bot }$ and $\varepsilon_{\parallel }$ as functions of BNA concentration at 1 kHz. (**d**) *Δε* and *Δn* as functions of BNA concentration. (**e**) *γ* at various BNA concentrations.

Figure 3a shows the V–T curves of the BNA-doped LC cells measured at RT. The curve shifted toward the low-voltage side as the BNA concentration increased, indicating that BNA doping decreased the operation voltage of the cell. A decrease in maximum transmission was obtained because BNA doping changed the refractive index of the LC mixture, resulting in refractive index mismatch between the interfaces or absorption of light by the BNA-doped LC mixture. The POM images of the BNA-doped LC cells with various voltages were measured to determine the threshold voltage (*V*_*th*_) of the cell. *V*_*th*_ was defined as the voltage at which the color of POM image began to change, indicating the initial distortion of LCs in the middle of the cell. As shown in Fig. 3b, *V*_*th*_ decreased with increased BNA concentration because BNA doping decreased *K*_*11*_ and increased *Δε* according to Eq. (1)^35–37^. Fig. 3c shows the changes in $\varepsilon_{\parallel }$ and $\varepsilon_{ \bot }$ with BNA concentrations at a frequency of 1 kHz. BNA doping increased the permittivity of the LC mixture.

1 $$ V_{th} = \pi \sqrt {\frac{{K_{11} }}{{\varepsilon_{0} \Delta \varepsilon }}} $$

As shown in Fig. 3d, BNA doping increased the *Δε* and decreased the *Δn* of the LC mixture. When the BNA concentration exceeded 1.5 wt%, *Δn* was saturated. The increment in *Δε* can be explained as follows. First, a strong polar terminal group (e.g., Cyano) usually causes a large *Δε*. BNA is a strong polar molecule such that BNA doping helps increase *Δε*. Second, BNA doping increases polarizability anisotropy (*Δα*) and hence *Δε*. Moreover, it enriches the short-range intermolecular forces. The presence of phenyl rings in BNA also changes the absorbance, *Δε*, *K*_*11*_, and *γ* of LC mixtures^37–39^. Generally, the accurate determination of elastic constant of LCs is less straightforward. The elastic constant is a parameter characterizing the elastic interaction between the LC molecules. When BNA is dispersed in LCs, the *K*_*11*_ of LC mixture also reflects the interaction between the LC molecules and the BNA dopant^40^. For the BNA-LCs composite, *K*_*11*_ can be roughly estimated by substituting *Δε* and *V*_*th*_ according to Eq. (1). Table 2 shows that BNA doping decreases *K*_*11*_ of the LC mixture. Moreover, BNA doping decreases the *T*_*NI*_ and hence the associated order parameter *S* and *Δn* of the LC mixture according to Eq. (2) and (3)^41–43^.

2 $$ S = (1 - T/T_{NI} )^{\upbeta } , $$

3 $$\Delta n =\Delta n_{0} (1 - T/T_{NI} )^{\upbeta } , $$

4 $$ \gamma { = }\left( {{\text{a}}_{0} {\text{ } + \text{ a}}_{1} {\text{S } + \text{ a}}_{2} {\text{S}}^{2} } \right)\exp \frac{{{\text{ES}}^{m} }}{{k_{b} \left( {T - T_{0} } \right)}}, $$

where *S* is the order parameter, *T* is ambient temperature, *T*_*0*_ is the melting point of the LC mixture, *m* is an exponent, *a*_*i*_ are proportionality, *Δn*_*0*_ is the birefringence of the LC mixture at 0 K, *k*_*b*_ is the Boltzmann constant, *β* is a material parameter, and *E* is the activation energy of molecule rotation. For many of the LC compounds studied, *β* are approximately 0.25 and insensitive to materials^44^.Eq. (2) is only valid for *T* sufficiently smaller than *T*_*NI*_^45,46^. The BNA molecule composed of biphenyl rings induced an interaction with the polar substituents of LCs and decreases *T*_*NI*_, *Δn* and *S*^37,39,47^. In this study, *T*/*T*_*NI*_ is less than 0.5 and thus *S* can be estimated by substituting *T* = 298 K, *β* = 0.25, and *T*_*NI*_ into Eq. (2). As shown in Table 2, with 3 wt% BNA doping, *S* decreased by ~ 5%. The decreased *S* also indicates the decrement in *K*_*11*_ because *K*_*11*_ is proportional to *S*^*2*^^48^. In addition, *γ* is proportional to *E* and *S*. Meier and Saupe reported that relaxation time $\tau_{\parallel }$ is related to the potential barrier parameter *η* in VA LC cells^49^.

5 $$ \tau_{\parallel } \sim \frac{\exp (\eta ) - 1}{\eta }, $$

where *η* can be estimated by substituting *S* into the equation^50^

6 $$ \eta \approx \frac{{{\text{3S(5 - }}\pi {\text{S)}}}}{{{\text{2(1 - S}}^{{2}} {)}}}. $$

**Table 2:** BNA concentration-dependent *K*_*11*_, *T*_*NI*_, and estimated *S* of LCs.

BNA concertation (wt%)	*K*_*11*_ (pN)	(*T*_*NI*_ °C)	*S*
0	11.49	63.5	0.582
0.5	10.39	62.5	0.578
1.0	9.60	61.0	0.573
1.5	9.68	59.5	0.568
2.0	8.69	58.0	0.562
3.0	6.92	55.5	0.552

In this experiment, the estimated *η* and $\tau_{\parallel }$ decreased with increased BNA concentration due to the reduced *S* (Table 2). *E* is related to $\tau_{\parallel }$ according to Eq. (7)^51^.

7 $$ E = 2.303 \, RT \, \log \left( {\frac{{\tau_{\parallel } k_{b} T}}{h}} \right), $$

where *R* is the molar gas constant, and *h* is the Plank constant. The decreased $\tau_{\parallel }$ reduced *E* with increasing BNA concentration. Consequently, BNA doping decreased *γ* due to the decrements in the *S* and *E* of the LC mixture. The *γ* of LC mixture is also experimentally determined by transient-current measurement^52^. As shown in Fig. 3e, *γ* is decreased by ~ 44% with the increased BNA concentration.

**Figure 4:**
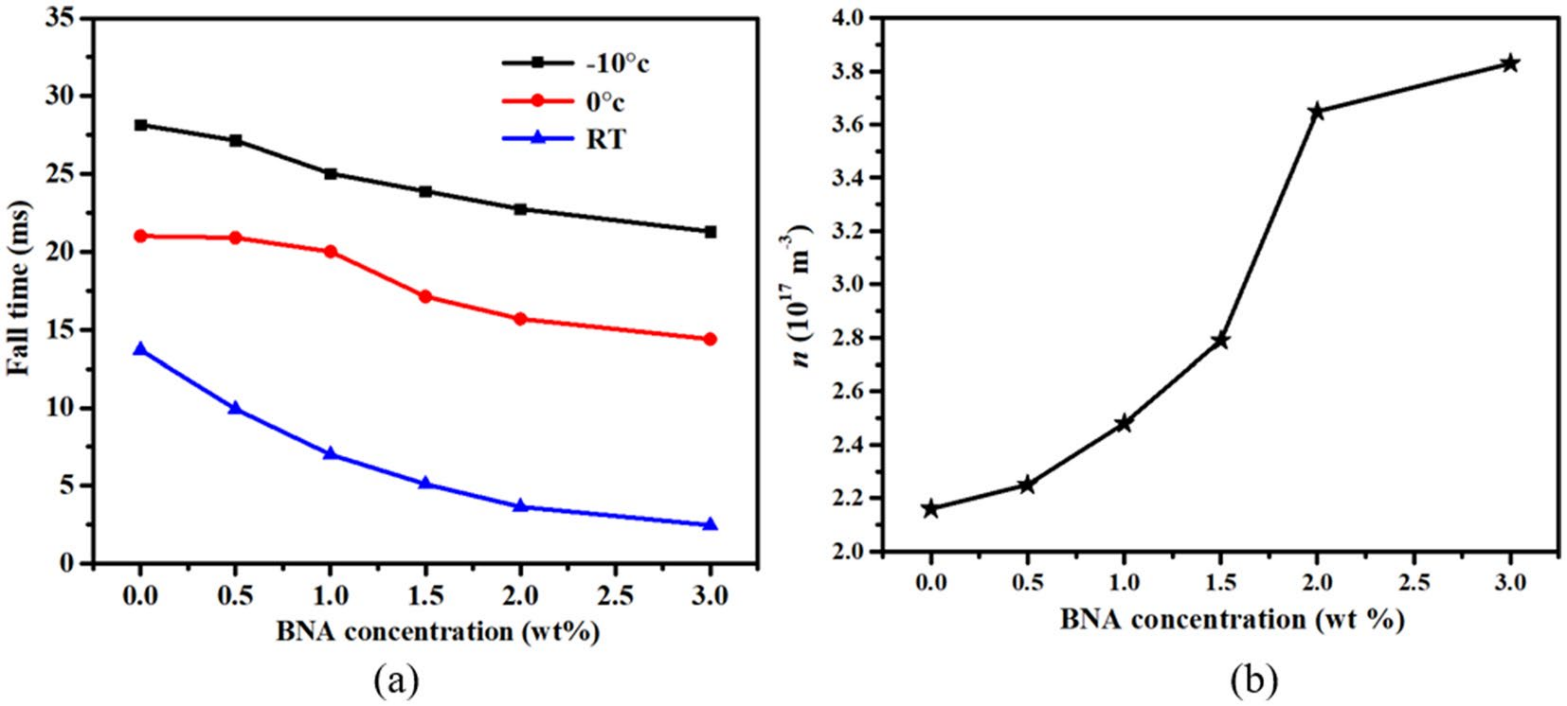
(**a**) Fall times of the BNA-doped LC cells at different temperatures. (**b**) *n* at various BNA concentrations.

Figure 4a shows the fall times of the BNA-doped LC cells at various temperatures. Rise (fall) time was defined as the time required for the transmission to change from 90 to 10% (10–90%) of the maximum transmission when the cell is turned on from 2 to 10 V (turned off from 10 to 2 V). Rise time is significantly smaller than fall time because of the former’s electric torque-driven reorientation, whereas the latter has a free relaxation reorientation. The rise times of the BNA-doped LC cells were almost constant at ~ 3.23, 1.58, and 0.67 ms for − 10°, 0°, and RT, respectively, due to the same turn-on voltage. Meanwhile, the fall time of the BNA-doped LC cells decreased with increased BNA concentration because BNA doping decreased the *γ* of the LC mixture and the *V*_*th*_ of the cell. Notably, the 3 wt% BNA-doped LC cell showed a fall time that was five times faster than that of the pure one at RT. If the applied voltage (*V*_*app*_) is much higher than *V*_*th*_, the relationships among fall time (*τ*_*off*_), rise time (*τ*_*on*_), *V*_*th*_, and *γ* can be expressed as follows:^43^

8 $$ \tau_{o} = \frac{{\gamma d^{2} }}{{K_{11} \pi^{2} }}, $$

9 $$ \tau_{on} = \frac{{\tau_{o} }}{{\left| {\left( {\frac{{V_{app} }}{{V_{th} }}} \right)^{2} - 1} \right|}}, $$

10 $$ \tau_{off} = \frac{{\tau_{o} }}{{\left| {\left( {\frac{{V_{bios} }}{{V_{th} }}} \right)^{2} - 1} \right|}}, $$

where *τ*_*0*_ is the relaxation time constant when the LC cell is turned off from *V*_*app*_ slightly higher than *V*_*th*_, *K*_*11*_ is the splay elastic constant, *V*_*bios*_ is the bios voltage, and *d* is the cell thickness. BNA doping significantly decreased *τ*_*off*_ due to the reduced *γ* and *V*_*th*_. Alkyl (methyl-CH_3_) and phenyl groups are known to decrease viscosity. BNA has a phenyl group, so BNA doping can reduce viscosity^47,53^.

The additional restoring force provided by the BNA’s SPEF also decreases the fall time of LC cell. If we consider BNA molecule as a dipole. The direction of the resultant dipole moment surrounding the BNAs (local regions) could be different from the director of LCs^54,55^. When no electric field is applied to the cell, the LCs near the local region orient along the resultant diploe moment direction, but other LCs still align parallel to the cell substrate. Consequently, BNA doping slightly disturbs the LC alignment and decreases the average *S* of the LC mixture. As a sufficient high electric field is applied to the cell, the LCs near in local regions as well as other regions reorient parallel to the applied electric field. Once the applied field is turned off, the LCs in local regions tend to return to their previously resultant dipole moment directions due to the BNA’s SPEF, creating stronger restoring force that significantly decreases the fall time of the cell.

The NO_2_ group in BNA makes the LC cell unstable, because of the increased ion density (*n*) in the LC cell. The increased *n* worsens the image sticking in the LC device. In this paper, the *n* of BNA-doped LC cell is determined by dielectric spectrum method^56^. As shown in Fig. 4b, as expected, the *n* increases from ${2}{\text{.16}} \times {10}^{{{17}}} \, m^{ - 3}$ to ${3}{\text{.83}} \times {10}^{{{17}}} \, m^{ - 3}$ with BNA concentrations. The doping of metallic/metal oxide NPs to suppress the ion density or operating the cell with high frequency is possible solution for the image sticking issue.

**Figure 5:**
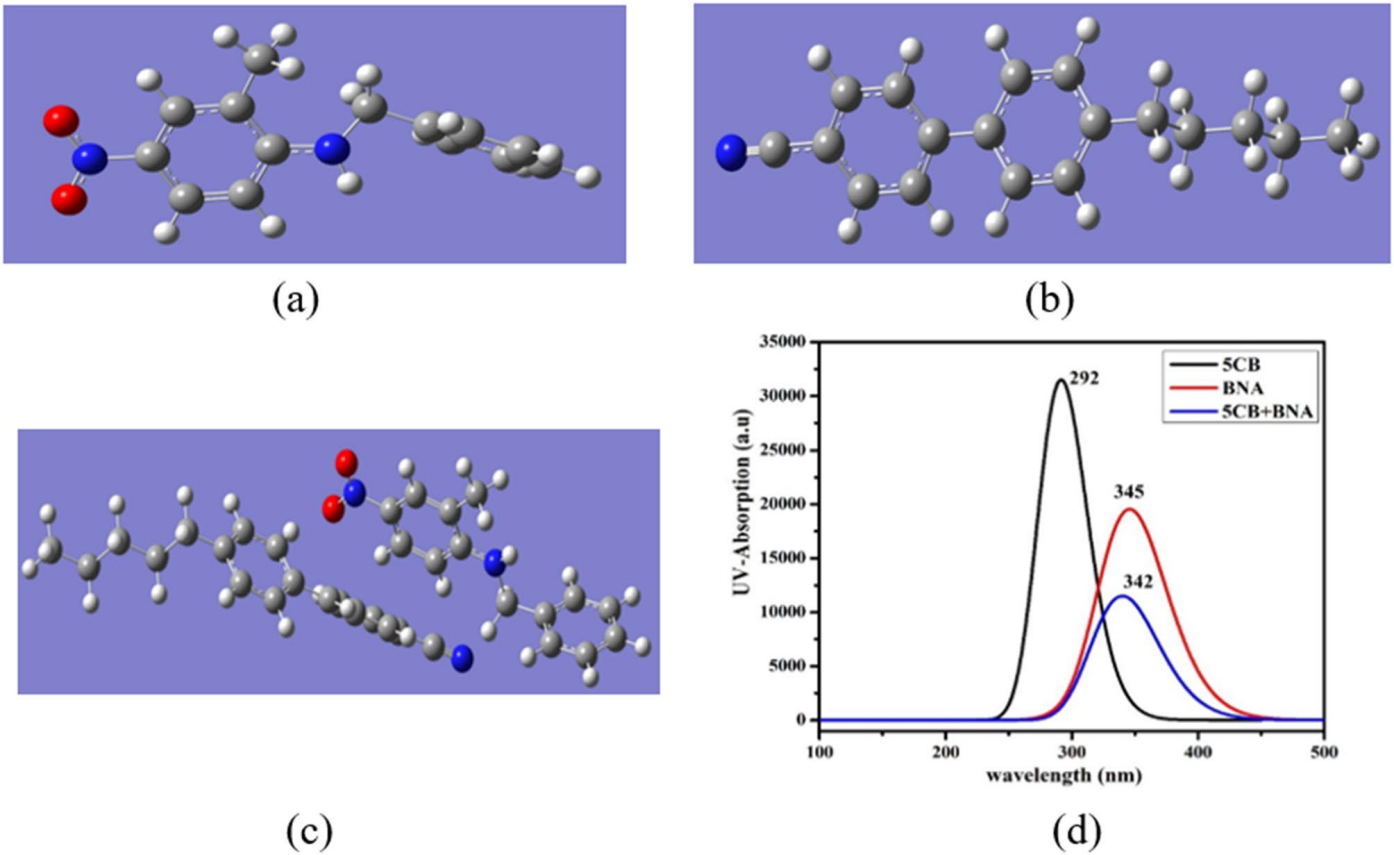
Geometry structures of (**a**) BNA, (**b**) 5CB, and (**c**) 5CB + BNA; (**d**) theoretical absorption spectra for BNA, 5CB, and BNA + 5CB.

DFT is a potential tool to explain the electronic structure of molecules. As shown in Fig. 5a–c, the structure optimization of aligned geometry between the BNA and 5CB molecule was performed using DFT with Becke-3–Lee–Yang–Parr (B3LYP) at the 6–31 + G (2d, p) basis set using Gaussian 09 software^57–60^. It was also used to explain the effects of the BNA-E7 mixture due to the monomer 5CB being the major component of LC E7. Several parameters, such as ultraviolet–visible (UV–vis) absorption spectrum, highest occupied molecular orbital (HOMO), lowest unoccupied molecular orbital (LUMO), molecular electrostatic potential, *Δε*, *Δα*, *μ*, and polarizability (*α*), were further obtained from the aligned geometry.

Figure 5d shows the calculated optical absorption spectra of 5CB, BNA, and 5CB + BNA, which provides an insight into the linear electronic absorption properties. The absorption peaks for 5CB and BNA appeared at 292 nm (π → π*) and 345 nm (n → π*), respectively, due to the presence of nitro and methyl groups^61^. The calculation results also indicated that BNA dopant shifted the absorbance wavelength of the LC cell toward 400 nm, confirming the partial contribution of BNA dopant to the yellowish CIE chromatographic coordinate of the BNA-doped LC cell, as shown in the inset of Fig. 2c.

The molecular orbital transition between HOMO and LUMO was calculated from the UV–vis absorption spectrum in Fig. 5d. HOMO acts as an electron donor, whereas LUMO acts as an electron acceptor. Here, HOMO and LUMO were concentrated entirely over anionic and cationic moieties, respectively. The HOMO–LUMO energy gaps (*ΔE*) of BNA and 5CB molecules was calculated using DFT with B3LYP at the 6–31 + G (2d, p) basis set using Gaussian 09 software. As shown in Table 3, the calculated *ΔE* of BNA, 5CB, and BNA + 5CB were 3.79, 4.60, and 3.91 eV, respectively^62^. A low value of the *ΔE* pertains to the concluding CT interactions taking place within the molecule and causes a highly polarized electronic structure. The relationship between dielectric constant and the *ΔE* is determined by^63^

11 $$\upvarepsilon (q) = 1 + \frac{1}{V}\frac{16\pi }{{q^{2} }}\sum\limits_{i}^{occ.} {\sum\limits_{a}^{vir.} {\frac{{\left| {\left\langle {\Phi _{i} (r)\left| {\exp (iq.r\left. ) \right|\Phi _{a} (r\left. ) \right\rangle \left| {^{2} } \right.} \right.} \right.} \right.}}{{E_{i} - E_{a} }}} } , $$

where the indices *i* and *a* represent the occupied and virtual (unoccupied) orbitals, respectively; *ε* is the dielectric constant; *q* is the wave vector; *E*_*i*_ and *E*_*a*_ are the orbital energies for occupied and virtual orbitals of $\phi_{i}$ and $\phi_{a}$, respectively; and *V* is the volume of the target molecule. *ΔE* is the difference between the occupied (*E*_*i*_) and virtual (*E*_*a*_) orbital energies, which appears in the denominator of Eq. (11). Therefore, a decreased *ΔE* increases dielectric constant *ε*. As shown in Table 3, BNA doping decreased *ΔE* and hence increased the permittivity ($\varepsilon_{\parallel }$ and $\varepsilon_{ \bot }$) of the LC mixture.

**Table 3:** Calculated *ΔE, μ*, *α*, and *Δα* of BNA and 5CB molecules.

Molecular geometry	Energy gap *ΔE* (eV)	*μ* (Debye)	*α* (a.u.)	*Δ*α (a.u.)
5CB	4.60	6.30	238.29	215.15
BNA	3.79	8.80	207.99	163.50
5CB + BNA	3.91	14.07	446.15	339.01

**Figure 6:**
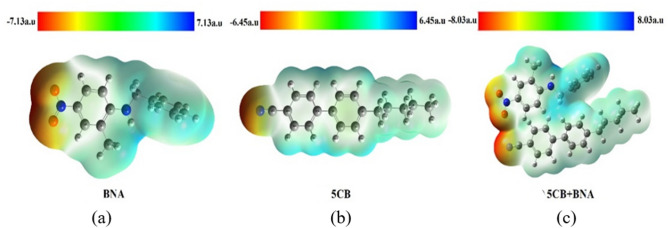
MEPs of (**a**) BNA, (**b**) 5CB, and (**c**) BNA + 5CB.

The molecular electrostatic potential (MEP) is related to the charge distributions of molecules and is calculated using DFT with B3LYP at the 6–31 + G (2d, p) basis set using Gaussian 09 software. MEP can be used to determine molecular interactions and analyse the bonding nature. MEP is related to the electronic density sites for electrophilic attacks and nucleophilic reactions as well as halogen and hydrogen-bonding interactions^64^. In Fig. 6, the negative (red) and positive (blue) regions represent electrophilic reactivity and nucleophilic reactivity sites, respectively. NO_2_ and the cyano groups are located in negative regions, whereas NH_2_ and CH_2_ are located in the positive region. MEP represents the net electrostatic effect of a molecule generated from the total charge distribution. It is extensively correlated with partial charges, electronegativity, dipole moments, and chemical reactivity. The total atomic electric dipole moment (*μ*) for the halogen bond participants in the charge-transfer complexes D_m_ …X–Y of the group was determined. The result showed that the magnitude (from − 8.03 to + 8.03 a.u.) of the total atomic dipole moment |*μ*_(D)_| was the largest for the BNA + 5CB complexes, indicating that it exhibited the highest interaction energy (*E*_*int*_)^65,66^. Consequently, BNA doping increased E_int_ and the dipole moment (*μ*) and significantly increased the permittivity of the LC mixture^67^, as shown in Fig. 3c.

The Maier–Meier equation was used to calculate *Δε* in consideration of the anisotropy in molecular polarizability and the orientation of the permanent dipole moment, as shown in Eqs. (12) - (14)^68^.

12 $$ \varepsilon_{\parallel } = 1 + \frac{NFh}{{\varepsilon_{0} }}\left\{ {\alpha { + }\frac{2}{3}\Delta \alpha S + \frac{{F\mu^{2} }}{{3k_{b} T}}(1 - (1 - 3\cos^{2} \theta )S} \right\}, $$

13 $$ \varepsilon_{{ \bot }} = 1 + \frac{NFh}{{\varepsilon_{0} }}\left\{ {\alpha { - }\frac{1}{3}\Delta \alpha S + \frac{{F\mu^{2} }}{{3k_{b} T}}(1 + \frac{1}{2}(1 - 3(\cos^{2} \theta )S} \right\}, $$

14 $$\Delta \varepsilon = \frac{NFh}{{\varepsilon_{0} }}\left\{ {\Delta \alpha { - }\frac{{F\mu^{2} }}{{2k_{b} T}}(1 - 3(cos\theta )^{2} )} \right\}S, $$

where *N* is the molecular number density; *ε*_*0*_ is the vacuum permittivity; *F* and *h* are constants of proportionality called the reaction field factor and the cavity factor, respectively; and *θ* is the dipole moment orientation angle relative to the long principal axis of the molecular frame. *α* and *Δα* can be calculated as

15 $$ \alpha = \frac{{\alpha_{xx} + \alpha_{yy} + \alpha_{zz} }}{3}, $$

16 $$ \Delta \alpha = \alpha_{xx} - \frac{{\alpha_{xx} + \alpha_{yy} }}{2}, $$

where *α*_*xx*_ is the molecular polarizability parallel to the molecular long principal axis and *α*_*yy*_ and *α*_*zz*_ are the molecular polarizabilities perpendicular to the molecular long principal axis.

As shown in Table 3, the *μ*, *α*, and *Δα* for 5CB, BNA, 5CB + BNA, and 5CB + BNA + 5CB were calculated by using DFT applying the B3LYP method with the 6–31 + G (2d, p) basis set of the Gaussian’09 software. The increments in *μ, α*, and *Δα* were more considerable than the decrement (only 5%) in *S* with BNA doping, indicating that the enhancement in *Δε* was mainly attributed to the increment in *μ, α*, and *Δα* according to Eqs. (12) - (14).

## Conclusions

The electro-optical effects of organic BNA dispersed in nematic LCs were demonstrated in this study. In the experiment, the BNA dopant preserved the color performance but slightly decreased the transmission of LC cells due to the refractive index mismatch between the interfaces and BNA absorbance. When the BNA-doped concentration reached 3 wt%, the *V*_*th*_ of the LC cells decreased by 25% due to the decreased *K*_*11*_ and increased *Δε* of the LC mixture. The *S* and *Δn* of the LC mixture decreased by ~ 5% due to the decreased *T*_*NI*_. Moreover, BNA doping decreased the activation energy *E* of the LC mixture. Consequently, BNA doping decreased the *γ* of the LC mixture. Notably, the 3 wt% BNA-doped LC cell had a fall time that was five times faster than that of the undoped LC cell, due to the decreased *γ* and *V*_*th*_, and the additional restoring force induced by the BNA’s SPEF. In the calculation, the BNA dopant increased the *E*_*int*_, *μ*, *α*, *Δα*, and *Δε* of the LC mixture and decreased its *ΔE*. The decreased *ΔE* increased the permittivity of the LC mixture. For practical applications, the optimal combination of LC and organic molecules still needs more in-depth research.
